# LIVER RETRANSPLANTATION: PROGNOSTIC SCORES AND RESULTS IN THE STATE OF PARANÁ

**DOI:** 10.1590/0102-672020240009e1802

**Published:** 2024-05-20

**Authors:** Alexandre Coutinho Teixeira de FREITAS, Israel Suckow GIACOMITTI, Vinicius Marques de ALMEIDA, Júlio Cezar Uili COELHO

**Affiliations:** 1 Universidade Federal do Paraná, University Hospital, Digestive Surgery Unit – Curitiba (PR), Brazil.

**Keywords:** Liver Transplantation, Liver Diseases, Survival Analysis, Risk Assessment, Transplante de Fígado, Hepatopatias, Análise de Sobrevida, Medição de Risco

## Abstract

**BACKGROUND:**

Hepatic retransplantation is associated with higher morbidity and mortality when compared to primary transplantation. Given the scarcity of organs and the need for efficient allocation, evaluating parameters that can predict post-retransplant survival is crucial.

**AIMS:**

This study aimed to analyze prognostic scores and outcomes of hepatic retransplantation.

**METHODS:**

Data on primary transplants and retransplants carried out in the state of Paraná in 2019 and 2020 were analyzed. The two groups were compared based on 30-day survival and the main prognostic scores of the donor and recipient, namely Model for End-Stage Liver Disease (MELD), MELD-albumin (MELD-a), Donor MELD (D-MELD), Survival Outcomes Following Liver Transplantation (SOFT), Preallocation Score to Predict Survival Outcomes Following Liver Transplantation (P-SOFT), and Balance of Risk (BAR).

**RESULTS:**

A total of 425 primary transplants and 30 retransplants were included in the study. The main etiology of hepatopathy in primary transplantation was ethylism (n=140; 31.0%), and the main reasons for retransplantation were primary graft dysfunction (n=10; 33.3%) and hepatic artery thrombosis (n=8; 26.2%). The 30-day survival rate was higher in primary transplants than in retransplants (80.5% vs. 36.7%, p=0.001). Prognostic scores were higher in retransplants than in primary transplants: MELD 30.6 vs. 20.7 (p=0.001); MELD-a 31.5 vs. 23.5 (p=0.001); D-MELD 1234.4 vs. 834.0 (p=0.034); SOFT 22.3 vs. 8.2 (p=0.001); P-SOFT 22.2 vs. 7.8 (p=0.001); and BAR 15.6 vs. 8.3 (p=0.001). No difference was found in terms of Donor Risk Index (DRI).

**CONCLUSIONS:**

Retransplants exhibited lower survival rates at 30 days, as predicted by prognostic scores, but unrelated to the donor’s condition.

## RESUMO

**RACIONAL:**

O retransplante hepático está associado a maior morbimortalidade do que o transplante primário. Dada a escassez de órgãos e a necessidade de alocação eficiente, avaliar parâmetros que possam prever a sobrevida pós-retransmissão é crucial.

**OBJETIVOS:**

Analisar os resultados dos retransplantes hepáticos em relação aos principais escores prognósticos.

**MÉTODOS:**

Foram analisados os transplantes primários e os retransplantes realizados no Estado do Paraná nos anos de 2019 e 2020. Os dois grupos foram comparados em relação à sobrevida em 30 dias e aos principais escores prognósticos do doador e do receptor: *Model for End-Stage Liver Disease* (MELD), *MELD-albumin* (MELD-a), Donor MELD (D-MELD), *Survival Outcomes Following Liver Transplantation* (SOFT), *Preallocation Score to Predict Survival Outcomes Following Liver Transplantation* (P-SOFT) e *Balance of Risk* (BAR).

**RESULTADOS:**

Foram incluídos 425 transplantes primários e 30 retransplantes. A principal etiologia da hepatopatia no transplante primário dos pacientes retransplantados foi o etilismo (n=140; 31,0%), e os principais motivos para os retransplantes foram o não funcionamento primário do enxerto (n=10; 33,3%) e a trombose da artéria hepática (n=8; 26,2%). A sobrevida em 30 dias foi maior nos transplantes primários em relação aos retransplantes (80,5% *vs* 36,7%; p=0,001). Os escores prognósticos foram mais elevados nos retransplantes em relação aos transplantes primários: MELD 30,6 *vs* 20,7 (p=0,001); MELD-a 31,5 *vs* 23,5 (p=0,001); D-MELD 1234,4 *vs* 834,0 (p=0,034); SOFT 22,3 *vs* 8,2 (p=0,001); P-SOFT 22,2 *vs* 7,8 (p=0,001); e BAR 15,6 *vs* 8,3 (p=0,001). Não foi observada diferença em relação ao Índice de Risco do Doador.

**CONCLUSÕES:**

Os retransplantes apresentam menor sobrevida em 30 dias, prevista nos escores prognósticos, porém sem relação com a qualidade dos doadores.

## INTRODUCTION

Retransplantation serves as the sole potential treatment for graft failure following primary liver transplantation^30^. However, this procedure is highly intricate and is associated with a poorer survival outcome compared with primary transplantation. As per the Organ Procurement and Transplantation Network and Scientific Registry of Transplant Recipients (OPTN/SRTR) Annual Data Report, the five-year survival rate was 12% lower for retransplantation^16^. Additionally, retransplantation is linked to a twofold increase in both intensive care unit (ICU) stays and costs^16^.

The indication for retransplantation varies based on the timeframe of the procedure. Shortly after primary transplantation, approximately 70% of graft losses occur due to primary non-function and vascular thrombosis^4^. Beyond one year, over 50% of losses are attributed to chronic rejection and the recurrence of viral hepatitis or other primary diseases^4^.

Zimmerman et al. identified several factors predicting higher mortality, including surgery performed 8–30 days after the primary transplantation, Model for End-Stage Liver Disease (MELD) score higher than 25, the need for respiratory support, advanced renal failure, and donor and recipient age^33^. There remains no consensus on whether the increased mortality associated with retransplantation is linked to the cause of graft failure or the underlying cause of cirrhosis^4^. Despite elevated mortality, retransplantation demonstrated favorable long-term survival in selected cases^32^.

The retransplant patient profile remains undetermined in Brazil, thus, underscoring the importance of exploring this aspect for more efficient organ allocation is a critical consideration given the shortage of donors^2^.

This study aimed to assess the outcomes of liver retransplantations conducted in the state of Paraná, Brazil, in 2019 and 2020, utilizing the main prognostic scores.

## METHODS

This is a multicenter, prospective, and retrospective study analyzing donors, primary cadaveric liver transplantations, and retransplantations in adults conducted in the state of Paraná during 2019 and 2020. Data were collected from the state Registry. Exclusion criteria consisted of the absence of a complete registry, living donor liver transplantation, and the inability to complete surgery on the recipient.

Patients were categorized into two groups: group 1 included patients submitted to primary liver transplantation, and group 2, those submitted to retransplantation. The following data were analyzed and compared between the two groups: donor age, recipient age, sex, ethnicity, 30-day patient survival, etiology of cirrhosis, cause of retransplantation, MELD score^14^, and MELD-a adjusted to MELD-sodium (MELD-Na) score^5^. Additionally, they were compared in relation to previously validated prognostic scores: D-MELD (Donor-MELD)^13^, Donor Risk Index (DRI)^12^, Balance of Risk Score (BAR)^7,11^, Score to Predict Survival Outcomes Following Liver Transplantation (SOFT)^23^, and Preallocation Score to Predict Survival Outcomes Following Liver Transplantation (p-SOFT)^23^.

Data were presented as mean values and standard deviations for numeric variables, and as absolute and relative frequencies for categorical variables. These variables were compared using Fisher’s Exact Test, Mann-Whitney test, and Chi-square test (χ^2^) with R Core Team 2022 software^22^.

The Ethics Committee of the University Hospital of Universidade Federal do Paraná approved this research under 62982722.6.0000.0096.

## RESULTS

In 2019 and 2020, a total of 468 liver transplantations were conducted in the state of Paraná: 438 (93.59%) were primary procedures and 30 (6.41%) were retransplantations. Within the primary procedure group, 13 patients were excluded due to incomplete registrations.

Table 1 displays a descriptive analysis and comparison of donor and recipient variables between the primary transplant and retransplant groups. The mean recipient age was higher in primary transplants than in retransplants (55 standard deviation [±]11 vs. 49±10, p=0.003). The age of the donors was comparable (41±15 vs. 40±14, p=0.321). There were more male than female patients in both primary transplants and retransplants: 69.9% (n=297) vs. 30.1% (n=128), (p=0.001); and 63.3% (n=19) vs. 36.7% (n=11), (p=0.001), respectively. No significant differences were observed according to the gender. The majority of patients were white in both groups: 76.5% (n=325) in primary transplant and 66.7% (n=20) in retransplants, and no significant differences were noted (p=0.132). No disparity was observed concerning cold ischemia time: 370 minutes in primary transplants and 335 minutes in retransplantation (p=0.1470).

**Table 1:** Descriptive analysis and comparison of donor and recipient variables between primary transplant and retransplant groups.

Variables	Total	Primary transplant	Retransplant	p-value
n (%)	455 (100)	425 (93.4)	30 (6.6)	
Mean age (years)	54±11	55±11	49±10	0.003
Donor mean age (years)	41±15	41±15	40±14	0.321
Gender n (%)				
Male	316 (69.4)	297 (69.8)	19 (63.3)	0.584
Female	139 (30.5)	128 (30.1)	11 (36.6)	
Ethnicity n (%)				
White	345 (75.8)	325 (76.4)	20 (66.6)	0.132
Black	26 (5.7)	22 (5.1)	4 (13.3)	
Brown	84 (18.4)	78 (18.3)	6 (20.0)	
Cold ischemia time (minutes)	368±122	370±123	335±117	0.147

±standard deviation.

Table 2 outlines the etiology of cirrhosis in the primary transplantation group and in those subjected to retransplantation. The primary transplantation group’s most common indications were alcoholic cirrhosis (30.8%), hepatocellular carcinoma (14.8%), and metabolic dysfunction-associated steatotic liver disease (11.0%). The most common etiologies of cirrhosis in primary transplantation for those who underwent retransplantation were alcoholic cirrhosis (n=9; 30.0%), viral hepatitis (n=4; 13.3%), and metabolic dysfunction-associated steatotic liver disease (n=4; 13.3%). In 20 cases, retransplantation was performed on an emergency basis 12±12 days after the primary procedure: in ten cases (33.3%) due to primary non-function, in eight (26.3%) due to hepatic artery thrombosis, and in two cases for other reasons. In ten cases, retransplantations were performed later, 1,035±961 days after the primary procedure. The reasons were chronic hepatic diseases: one (3.3%) autoimmune hepatitis, two (6.7%) cryptogenic cirrhosis, one (3.3%) secondary biliary cirrhosis, two (6.7%) alcoholic cirrhosis, and four (20.0%) were designated as other etiologies.

**Table 2:** General etiology of chronic hepatopathy, etiology of chronic hepatopathy in retransplant group and retransplant indications.

General chronic hepatopathy etiology	n (%)	Retransplant chronic hepatopathy etiology	n (%)	Indications for retransplant	n (%)
Alcohol	140 (30.8)	Alcohol	9 (30.0)	Primary non-function	10 (33.3)
HCC	67 (14.8)	Viral hepatitis	4 (13.3)	Hepatic artery thrombosis	8 (26.2)
MAFLD	50 (11.0)	MAFLD	4 (13.3)	Alcohol	2 (6.6)
HBV	33 (7.2)	AIH	3 (10.0)	Cryptogenic cirrhosis	2 (6.6)
HCV	22 (4.8)	Cryptogenic cirrhosis	3 (10.0)	AIH	1 (3.3)
AIH	20 (4.4)	HCC	2 (6.6)	SBC	1 (3.3)
Others	123 (27.0)	PSC	1 (3.3)	Others	6 (20)
		PBC	1 (3.3)		
		SBC	1 (3.3)		
		Others	2 (6.6)		

HCC: hepatocellular carcinoma; MAFLD: metabolic dysfunction-associated fatty liver disease; HBV: hepatitis B virus cirrhosis; HCV: hepatitis C virus cirrhosis; AIH: autoimmune hepatitis; PSC: primary sclerosing cholangitis; PBC: primary biliary cholangitis; SBC: secondary biliary cholangitis.

Table 3 illustrates the 30-day survival and scores predicting survival analysis. The 30-day survival was higher in the primary transplant group (80.5% vs. 36.7%; p<0.001). All predictors of survival scores were higher in patients submitted to retransplantation: MELD was 30.6±9.2 vs. 20.7±7.9 (p<0.001), respectively; MELD-Na was 31.5±9.2 vs. 23.5±7.2 (p<0.001); D-MELD was 1,234.4±558.8 vs. 834.0±415.0 (p=0.034); SOFT was 22.3±10.9 vs. 8.2±6.2 (p<0.001); P-SOFT was 22.2±10.2 vs. 7.8±5.7 (p<0.001); and BAR was 15.6±5.5 vs. 8.3±4.1 (p<0.001). The DRI score, considering only donor data, showed no difference between the groups: 1.4±0.3 vs. 1.4±0.4 (p=0.801).

**Table 3:** Comparison of 30-day survival, survival prediction scores, and postoperative complications between primary transplant and retransplant groups.

Variables	Primary transplant (n=425)	Retransplant (n=30)	p-value
30-day survival	80.4%	36.6%	0.001
MELD	20.7±7	30.6±9	0.001
MELD-a	23.5±7	31.5±9	0.001
D-MELD	834.0±415	1234.4±558.8	0.034
SOFT	8.2±6	22.3±10	0.001
P-SOFT	7.8±5	22.2±10	0.001
BAR	8.3±4	15.6±5	0.001
DRI	1.4±0.3	1.4±0.3	0.801

±standard deviation; MELD: Model for End-Stage Liver Disease, MELD-a: MELD-albumin, D-MELD: Donor MELD; SOFT: Survival Outcomes Following Liver Transplantation; P-SOFT: Preallocation Score to Predict Survival Outcomes Following Liver Transplantation; BAR: Balance of Risk; DRI: Donor Risk Index.

## DISCUSSION

In the present study, 6.4% of all liver transplants performed in the state of Paraná, required retransplantation, consistent with findings reported in other countries. Alamo et al. related retransplantation incidences ranging from 6.0 to 11.0%^1^, and Yoon et al. indicated a range of 5.5 to 7.0%^31^. In Canada, the rate was 6.5%^31^; in Poland, 6.3%^20^; in Germany, 9.2%^17^; in China, 4.3%^9^; and in Spain, rates varied between 6.3^26^, 8.4^3^, and 6.6%^1^.

Within the retransplantation group, the primary causes of cirrhosis in the initial transplantation were alcohol, hepatitis B and C viruses, and metabolic dysfunction-associated steatotic liver disease. Similar findings were observed by Lang et al.^17^, where hepatitis C, alcohol, and hepatitis B were the leading causes. Alamo et al.^1^ also identified alcohol, hepatitis C, and hepatitis B as common causes.

The prevalent indications for retransplantation were primary non-function and hepatic artery thrombosis, consistent with Lang et al.^17^. Masior et al.^20^ highlighted vascular complications as the primary cause, with rejection and primary non-function as the second and third causes. Torres-Quevedo et al.^26^ identified hepatic artery thrombosis, hepatitis C recurrence, and primary non-function as the primary reasons.

This study assessed the impact of prognostic factors on 30-day patient mortality, directly reflecting surgical risk. Prognostic scores are crucial for rational organ allocation decisions. Ethical considerations arise regarding transplantation with indicative of poor prognosis, given the scarcity of donors and mortality during the waiting list^15,18^.

Several authors demonstrated a poorer prognosis for retransplantation compared to primary transplantation. Berumen et al. reported 83.0, 75.0, and 69.0% survival rates at one, three, and five years on primary transplants, respectively, and 67.0, 60.0, and 53.0% on retransplants^4^, Yoon et al. showed 91.4, 86.0, 81.8, and 72.9% survival rates at one, three, five, and ten years on primary transplants, respectively, and 77.1, 70.4, 65.5, and 60.0% on retransplantations^31^. Masior et al. reported a retransplantation survival of 69.9% in the immediate postoperative period^20^.

In this study, 30-day patient survival after retransplantation was shorter than mentioned by other authors. In Brazil, patient survival post-primary transplant is already short compared to Europe and the United States. This discrepancy reflects donor care quality in ICUs, healthcare providers, including surgeons not exclusively dedicated to transplantations, lack of sophisticated equipment and technology, transportation logistics, time on the waiting list, and other factors.

It must also be considered that the majority of retransplants were performed on an emergency basis, typically a few days after the primary procedure. The major indications were primary non-function and hepatic artery thrombosis. This is correlated with a worse prognosis due to the critical condition of the patients^9,11,29^. The time interval between the primary procedure and retransplantation is also crucial. The shorter the time interval, the better the results. This aspect was not evaluated in the prognostic scores used in this study. The mean time interval was 12 days, which is longer than observed in the United States and Europe, where organ procurement is faster.

A study assessed 70 patients undergoing retransplantation, revealing a 57% survival rate when the procedure was conducted within three days after primary surgery and a 24% survival rate when performed between 4–30 days. Additionally, the same study demonstrated an 83% survival rate when retransplantation took place one year or more after the initial surgery^19^. Similar findings were reported by other authors^5,10,21,24,33^.

In our study, retransplanted patients had lower ages. However, this is not associated with an increased risk or need for retransplantation. On the contrary, older patients face an increased risk. Other epidemiological variables, such as gender and ethnicity, were similar between the groups.

Donor evaluation plays a crucial role in the decision-making process for organ allocation. The donor’s age serves as a key objective parameter in this assessment^28^. In our study, the donor’s age was similar when comparing primary transplantations and retransplantations. Consequently, it can be excluded as an indicative factor of the observed worse prognosis observed in retransplants. This finding is particularly significant since, as previously mentioned, the majority of retransplants were performed in emergency situations, immediately after the primary procedure. This scenario is considered a priority over other patients on the waiting list; the sooner an organ is made available the better the prognosis. Therefore, it might be feasible to consider marginal older donors as a potential solution to address this issue.

The DRI was also similar between the groups in our study. This score is composed of several variables^12^, with two of them carrying more weight in the score composition and were not present in our donors: donation after cardiac arrest and split liver transplantation. The other variables include donor age, brain death cause, and cold ischemia time. The last two variables were also similar between the two groups analyzed. Therefore, we can propose that marginal donors were not utilized, and donor condition did not interfere with retransplant patient mortality when compared to the primary procedure.

To study organ allocation based on donor and recipient condition, D-MELD was evaluated. It is calculated by multiplying donor age and recipient MELD score^13^. In other words, both donor and recipient factors influence the score. Donor age was not different between the two groups analyzed in this study. Therefore, the adverse clinical condition of the retransplant recipient, reflected in a higher MELD score in this group, also led to a higher D-MELD score. The same pattern was observed with MELD-Na, indicating the worst clinical condition of retransplant recipients. Nevertheless, MELD and MELD-Na are not as precise in indicating mortality risk after the procedure. These two scores predict mortality risk while on the waiting list^8^. This is the reason why these scores are used to prioritize the waiting list for primary transplantation in most countries.

BAR, SOFT, and P-SOFT scores were also correlated with the worst prognosis observed in retransplantation patients. These scores have been previously validated in the state of Paraná as prognostic indicators^25^. According to the authors of this study, values equal to or greater than 12 indicate higher mortality in all three scores. BAR has also been validated in two other studies, with better cutoff points identified as 9 and 11 points, respectively^6,7,27^. Other authors have also validated the SOFT score, with better cutoff points identified as 12 and 15 points^11,27^. In our study, in contrast to primary transplantation, retransplants presented with BAR, SOFT, and P-SOFT scores exceeding all these worst prognostic cutoff values.

The BAR score comprises MELD, recipient age, donor age, the need for life support, cold ischemia time, and the need for retransplantation^11^. Considering this, the MELD score, the need for life support, and the need for retransplantation were responsible for higher BAR scores and increased mortality observed in patients undergoing retransplantation. No difference was observed in the other score factors compared to primary transplantation. The recipient age was lower in the retransplantation group, and it even tended to reduce the score value by this parameter. BAR score is not suitable for use at the moment of organ allocation because cold ischemia time is a parameter obtained only after the procedure on the recipient is ongoing. Therefore, BAR does not permit the anticipation of the risk.

P-SOFT and SOFT are complementary scores developed by OPTN from 21,673 transplantations performed in the United States^23^. P-SOFT is composed of 14 recipient parameters that can be determined at the time of organ allocation. Among all the scores analyzed in this study, P-SOFT is the only one that allows for more efficient organ allocation in the case of retransplantation. Consequently, it enables the discerning of situations with prohibitive risk and potential for futile transplantation. The drawback of this score is that it does not consider donor factors. Nevertheless, it can be addressed by the transplantation team. SOFT comprises 22 parameters: 14 from P-SOFT, bleeding as a consequence of portal hypertension within 48 hours before transplantation, and six additional parameters related to the donor, including cold ischemia time. Due to this, similar to the BAR score, SOFT is not suitable for use during the process of organ allocation. Despite this limitation, SOFT was identified as the best prognostic score when compared to BAR and DRI in a study conducted in the state of Paraná^27^.

Determining the appropriate survival parameter to identify a futile transplantation is a complex issue. One approach suggests that transplantation survival should exceed survival while on the waiting list^23^. The survival probability for a patient in need of retransplantation depends on the timing of this procedure relative to the primary surgery. When retransplantation is required immediately after primary surgery, the mortality while on the waiting list approaches 100% if the procedure is not performed. The challenge lies in those patients in very poor clinical conditions with extremely high surgical risk. In such cases, it should be considered organ shortage and waiting list mortality for those patients. This is a valid argument to discourage retransplantation. However, these patients are generally in an acceptable clinical condition to proceed with retransplantation hours or just a few days after the primary procedure. It is important to note that this situation can deteriorate rapidly, and the decision to remove the patient from the waiting list is complex and challenging. Retransplantations months or years after the primary procedure are performed for chronic rejection or recurrence of the primary disease. In this situation, waiting list mortality is similar or slightly diminished compared to that expected for primary transplantation. Additionally, survival after retransplantation in this scenario is higher than retransplantation performed immediately after the primary procedure, making the decision less problematic^19^.

## CONCLUSIONS

Retransplantations exhibited lower survival than primary transplants, indicating a more unfavorable clinical condition as reflected in the following prognostic scores: BAR, P-SOFT, and SOFT. The evaluation of donor’s clinical condition by DRI did not influence retransplantation mortality.
